# Splenic abscess caused by *Streptococcus anginosus* bacteremia secondary to urinary tract infection: a case report and literature review

**DOI:** 10.1515/med-2020-0117

**Published:** 2020-10-08

**Authors:** Hao Wu, Rui Zheng

**Affiliations:** Department of Pulmonary and Critical Care Medicine, Shengjing Hospital of China Medical University, No. 36, Sanhao Street, Heping District, Shenyang, Liaoning 110004, China

**Keywords:** *Streptococcus anginosus*, bacteremia, splenic abscess, urinary infection, treatment

## Abstract

Organ abscesses caused by *Streptococcus anginosus* are relatively rare. We report the case of an elderly woman with splenic abscess caused by *S. anginosus* bacteremia after urinary tract infection. An 82-year-old woman had a history of frequency of urination, urgency, and fever with chills for over 10 days prior to admission. An abdominal computed tomography (CT) scan performed in the emergency room revealed a low-density lesion in the spleen, kidney cysts, some exudation around the kidney, and cystitis should be valued. She was treated with ceftriaxone and imipenem/cilastatin. After admission, the blood culture yielded positive results for *S. anginosus*. A contrast-enhanced abdominal CT scan showed that the low-density lesion previously found in the spleen was smaller than before. After percutaneous drainage of the splenic abscess and treatment with piperacillin/tazobactam based on the antibiotic sensitivity pattern, repeated abdominal CT scan revealed a significant reduction in the low-density lesion. The patient was discharged without recurrence or complications. A systematic review of organ abscess caused by *S. anginosus* bacteremia was performed. To our knowledge, there has been no report of splenic abscess caused by *S. anginosus* bacteremia secondary to urinary system tract infection, although urinary tract infections are also an important source.

## 1 Introduction

Urinary tract infection is the most common parenteral infection among females worldwide. One-third of women experience symptomatic urinary tract infections by the age of 24 years, and more than 50% of women will experience it during their lifetime [1]. The reduction in estrogen secretion that occurs in menopausal and postmenopausal women contributes to the transition of the dominant vaginal flora from *Lactobacillus* to *Escherichia coli* or other Enterobacterales, and increases the risk of urinary tract infection [1]. We present the case of an 82-year-old female patient who presented with a urinary tract infection, and in whom *S. anginosus*, part of the normal flora of the urinary tract, invaded the circulation, leading to splenic abscess formation. We review the literature on organ abscess caused by *S. anginosus* bacteremia and emphasize that different sources of infection should be considered.

## 2 Case presentation

An 82-year-old woman was admitted to our hospital with a chief complaint of recurrent fever for more than 10 days. Ten days prior, the patient experienced frequency of micturition, urgency, and fever with chills with no obvious predisposition; the highest body temperature was as high as 39.5°C. She had no odynuria. She self-treated with antipyretics. Three days prior, the patient experienced fever again, with a body temperature of 40°C, accompanied by chills, unconsciousness, fatigue, and nausea, but no vomiting. She was admitted to the emergency room of our hospital and treated with intravenous ceftriaxone. Antibiotic therapy was escalated to imipenem/cilastatin due to recurrent fever that did not respond to ceftriaxone at a dose of 2.0 g once a day for two days, and the body temperature reduced slightly. The patient had lower abdominal pain, but no diarrhea, dizziness, or headache. The patient had been retired from her job for many years and had no history of smoking or drinking in the past. She had no significant medical or family history.

Physical examination after admission indicated a body temperature of 36°C, pulse rate of 63 bpm, respiratory rate of 18 breaths per minute, and blood pressure of 120/51 mmHg. The breath sounds were coarse, and no rhonchi or moist rales could be heard. Cardiac examination revealed no murmurs. Upon abdominal examination, there was mild tenderness in the lower abdomen with no rebound tenderness or guarding. No other obvious abnormalities were observed.

**Figure 1:**
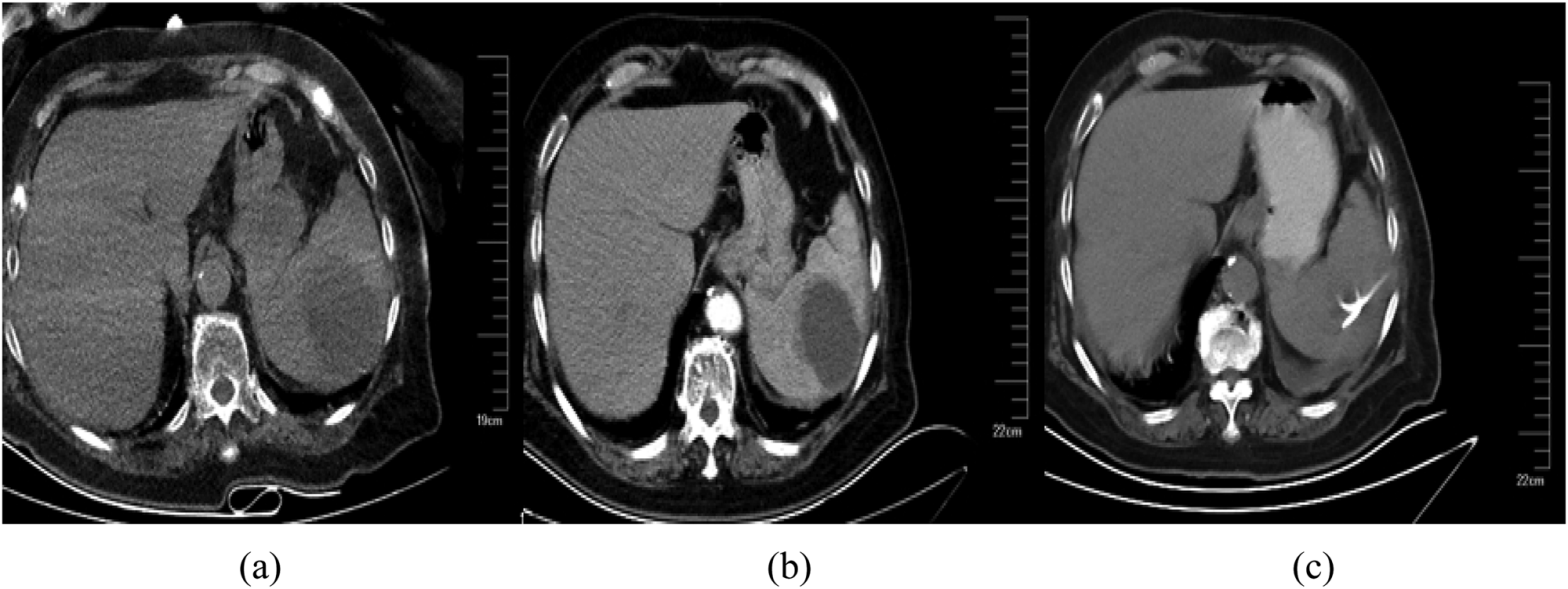
A CT scan of the abdomen revealed a low-density lesion in the spleen (a) before admission, (b) on day 5 after admission, (c) after paracentesis and drainage of the splenic abscess.

**Figure 2:**
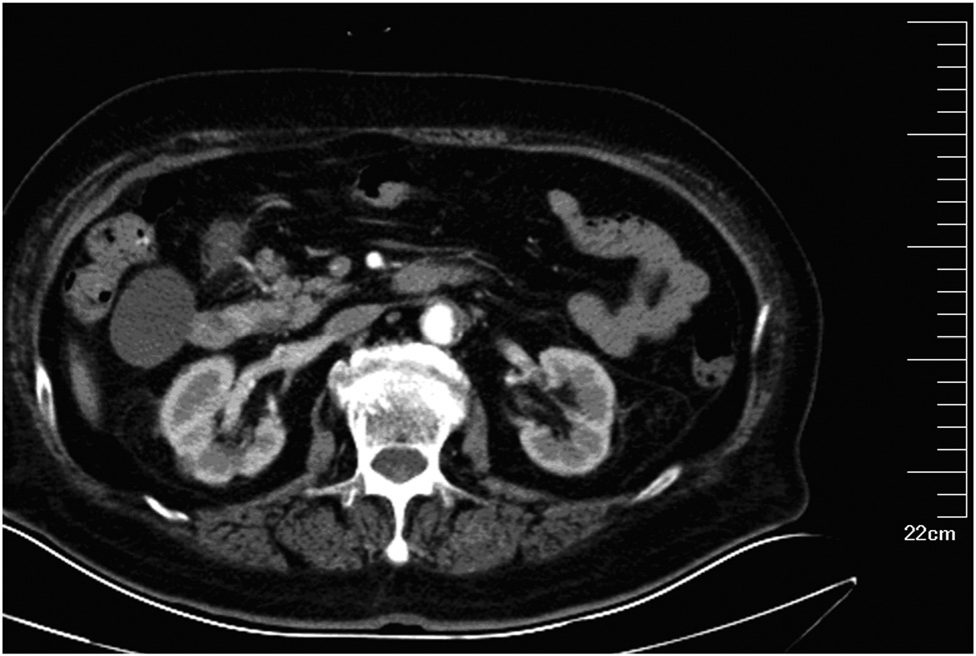
An abdominal CT scan showed kidney cysts and a little exudation around the kidneys.

**Figure 3:**
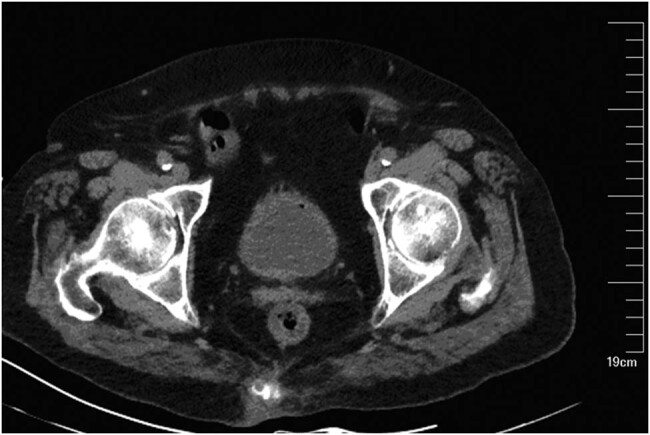
An abdominal CT scan showed thickening bladder wall and a little gas in the bladder.

Laboratory examination performed in the emergency room indicated the following: a white blood cell (WBC) count of 16.3 × 10^9^/L; neutrophils, 90.4%; and C-reactive protein level, 78.3 mg/L. A computed tomography (CT) scan of the lungs indicated subpleural patches in the lower dorsal lobe, considering for a sagging effect. A CT scan of the abdomen revealed splenomegaly, a low-density lesion in the spleen (Figure 1(a)), kidney cysts, a little exudation around the kidney (Figure 2), thickening of the bladder wall, a little gas in the bladder, and cystitis could not be ruled out (Figure 3). After admission, the procalcitonin level was 1.67 ng/mL; urine routine examination showed 1.7 WBCs/high-power field, red blood cell count 5.7/high-power field, and a bacterial count of 667/µL; and the albumin level was 32 g/L. Ultrasonography of the hepatobiliary tree and spleen revealed a cystic mass in the spleen. Echocardiography revealed aortic valve degeneration, aortic sclerosis, and normal left ventricular systolic function at rest.

After admission, the patient received imipenem intravenously at a dose of 0.5 g every 6 h, and the body temperature dropped significantly. The blood culture yielded positive results for *S. anginosus*. On day 5 after admission, a contrast-enhanced abdominal CT scan showed splenomegaly, and a smaller low-density lesion in the spleen than before, with multiple peripheral honeycomb-like weakly enhanced low-density lesions, indicative of splenic abscess (Figure 1(b)). The kidney cysts and exudation around the kidney were nearly the same as before. Percutaneous drainage was performed, and *S. anginosus* was isolated from the pus sample. Next, a drug susceptibility test was performed with bacterial suspensions by using the PMCI/ID-2 card matched by Phoenix 100 (BD Company, America) or disk diffusion method. The interpretation of antimicrobial susceptibility was performed by applying clinical breakpoints defined by the Clinical Laboratory Standards Institute M100-S29. The result showed that *S. anginosus* in the blood and pus was sensitive to penicillin G. And the antibiotics were changed to intravenous 80 piperacillin/tazobactam at a dose of 4.5 g every 8 h (Table 1).

**Table 1:** The susceptibility results of *S. anginosus*

Antibiotic name	Method	Sensitivity	Result	Determination standard		
				Sensitive	Intermediary	Resistance
Cefotaxime	MIC	S	≤0.5	≤1	2	≥4
Chloramphenicol	MIC	S	4	≤4	8	≥16
Cefepime	MIC	S	≤0.5	≤1	2	≥4
Clindamycin	MIC	R	>1	≤0.25	0.5	≥1
Erythromycin	MIC	R	>4	≤0.25	0.5	≥1
Linezolid	MIC	S	≤1	≤2	—	—
Vancomycin	MIC	S	1	≤1	—	—
Penicillin G	MIC	S	0.125	≤0.12	0.25–2	≥4

On day 16 after admission, re-examination via the abdominal CT scan revealed changes after paracentesis and drainage of the splenic abscess, and the low-density lesion had significantly reduced in size. A little gas could be seen in and around the spleen (Figure 1(c)). The levels of C-reactive protein and procalcitonin were 39.1 mg/L and 0.116 ng/mL, respectively. On day 22 after admission, the patient was discharged without recurrence or complications.


**Ethical approval:** The research related to human use has been complied with all the relevant national regulations, institutional policies, and in accordance with the tenets of the Declaration of Helsinki, and has been approved by the authors’ institutional review board or equivalent committee.
**Informed consent:** Informed consent has been obtained from all individuals included in this study.

## 3 Discussion

Urinary tract infections are the most common parenteral infections in women worldwide, especially among the elderly. Among outpatient female patients with symptoms of urinary tract infection such as dysuria and frequency of micturition without vaginal discharge, the incidence of cystitis is 96% [2]. Studies have shown that *E. coli* is the most common pathogen of urinary tract infection in the elderly. García-Agudo et al. also found that *E. coli* is the most common pathogen in community-acquired urinary tract infection, followed by *Klebsiella pneumoniae*, *Enterococcus faecalis*, *Pseudomonas aeruginosa*, and *Proteus mirabilis* [3], and the blood culture result was positive in 15–25% of urinary tract infection patients [4]. Krystal et al. confirmed that *S. anginosus* was one of the pathogens of genitourinary infection [5]. However, no study has reported that *S. anginosus* invading the blood after a urinary infection and resulting in a splenic abscess. In our case, the patient had symptoms of urinary tract infection such as frequency of micturition and urgency, and an abdominal CT scan indicated cystitis. The levels of bacteria and red blood cells were high in the urine. The normal WBC level in the urine may have been explained by the antibiotic treatment received before admission. It was a great pity that we neglected to perform a urine culture test because the symptoms of frequent urination and urgency disappeared after admission. Our patient was an elderly woman with a low albumin level, poor nutritional status, and decreased immunity. The colonization of the genitourinary system by *S. anginosus* could cause urinary tract infection, and lead to splenic abscess after entering the circulation. Thus, when urinary tract infection is suspected, we should be alert to the possibility of *S. anginosus* infection. Active treatment of the primary disease, early intervention, and effective prevention of bacteremia can control or prevent the occurrence or progression of the abscess.

The *S. anginosus* group comprises *S. anginosus*, *Streptococcus intermedius*, and *Streptococcus constellatus* [6], which are normal flora of the oral cavity, upper respiratory tract, gastrointestinal tract, and urogenital tract [7,8]. Epidemiological data show that *S. anginosus* can cause mild skin infection and even suppurative life-threatening infection [9]. The distribution and incidence of pyogenic infection are different among *S. anginosus*, *S. intermedius*, and *S. constellatus*. Studies have confirmed that *S. anginosus* is more likely to be isolated from the gastrointestinal tract and urogenital system, while *S. intermedius* is more possible to cause the infection of the central nervous system. *S. constellatus* can be isolated more widely, relatively more common in the respiratory tract, but less common in the central nervous system [10]. Moreover, *S. intermedius* has the tendency to form abscesses and infection in deep tissue and is more likely to cause suppurative non-bacterial infection, which requires surgical intervention. In contrast, *S. constellatus* and *S. anginosus* have a lower incidence of pyogenic infection [11].

Some studies have shown that *S. anginosus* can be associated with head, neck, and esophageal cancer [12]. Other studies have concluded that the most common source of *S. anginosus* bacteremia is the hepatobiliary system [13]. In a study of 127 cases of cancer, Sasaki et al. concluded that *S. anginosus* bacteremia was more common in esophageal and gastric cancer among patients with solid tumors complicated by *S. anginosus* group bacteremia [14]. This may be accounted for by the increasing amount of *S. anginosus* in the stomach when gastric tumors occur [15].

Organ abscesses caused by *S. anginosus* bacteremia are relatively rare, with only nine cases in the literature (Table 2). Almost all cases presented fever and abdominal pain and three cases showed nausea and vomiting. Most cases presented abdominal tenderness. Six cases had a gastrointestinal focus among which three had tumors of the digestive tract. Two cases had an oropharyngeal focus, and the patients had poor oral hygiene or suffered from dental caries and oral mucosal ulcers. Only one case had a biliary focus. The main mechanism underlying those with a digestive tract focus is that gastrointestinal tumors or invasive surgeries destroy the mucosa, offering an opportunity for the normal flora *S. anginosus* to invade the blood and cause abscesses in other parts of the body. The male/female ratio of these nine cases was 6:3; the mean age was 51 (range 32–63) years. Two patients experienced improvement after conservative intravenous medication without invasive procedures, and the other patients were treated with additional abscess drainage or surgery. Most patients had a good prognosis after intravenous medication and operation if necessary, and only two patients died (mortality rate, 22.2%), both of whom had abscesses involving the mediastinum, and one of them had tumor metastasis. Therefore, when mediastinal abscesses occur, treatment is relatively difficult, and early active intervention should be taken to reduce the mortality. In addition, *S. anginosus* bacteremia can also contribute to other infections without abscess formation, such as infective endocarditis [16], osteomyelitis [17], gas gangrene [18], endogenous endophthalmitis [19], and acute glomerulonephritis [20].

**Table 2:** Published cases of *S. anginosus* bacteremia leading to abscess formation

Ref.	Gender	Age (years)	Underlying diseases	Invasive pathway	Organ with abscess	Number of abscesses	Treatment of abscess	Outcome
Prashanth Rawla et al. [23]	Male	62	Hypertension, dyslipidemia, hypothyroidism,colon cancer	Cancer destroyed the integrity of the blood-mucosal barrier and led to the *S. anginosus* bacteremia	Liver	Single	Empirical antibiotics (vancomycin, metronidazole, piperacillin-tazobactam). Positive blood culture (ceftriaxone)	Improved
Umair Masood et al. [24]	Male	62	Hypertension, diabetes mellitus, colorectal cancer	Cancer destroyed the colonic mucosa, and the pathogens invaded the circulation	Liver	Multiple	Empirical antibiotics (vancomycin, piperacillin-tazobactam). Positive blood culture (ceftriaxone)	Improved
Marina Gorelik et al. [25]	Female	63	Hypertension, diabetes mellitus, ileal stromal tumors	Cancer destroyed the colonic mucosa, and the pathogens invaded the circulation	Liver	Multiple	Ceftriaxone, metronidazole, percutaneous drainage	Improved
Miguel E. Cervera-Hernandez et al. [26]	Female	45	Hypertension, asthma, morbid obesity status	Laparoscopic sleeve gastrectomy destroyed the colonic mucosa, and the pathogens invaded the circulation	Spleen	Single	Empirical antibiotics (piperacillin-tazobactam). Positive blood culture (meropenem, linezolid, ceftriaxone, metronidazole), percutaneous drainage	Improved
Daniel C Baumgart [27]	Male	41	Ulcerative colitis, fulminant pancolitis	Inflammatory bowel disease caused bacterial translocation of the gut, resulting in bacteremia	Endocardium		Empirical antibiotics (gentamicin, vancomycin), surgery	Improved
J Campos et al. [28]	Male	53	Hepatocarcinoma	Oropharyngeal infection	Peritonsillar abscess with adrenal and bone extending to the right posterior neck space and to the mediastinum		Piperacillin-tazobactam, imipenem, linezolid, fluconazole	Died
Huseyin Agah Terzi et al. [29]	Male	32	Poor oral hygiene, dental caries, impairment of mucosal integrity	Poor oral hygiene caused abscess and bacteremia	Abdominal cavity	Multiple	Ceftriaxone, metronidazole	Improved
Jia Dong et al. [30]	Female	41	Pneumonia		Mediastinum		Ciprofloxacin, clindamycin, moxifloxacin, etimicin, oseltamivir phosphate, linezolid	Died
Norikazu Arakura et al. [31]	Male	63	Acute obstructive suppurative cholangitis		Round ligament of the liver		Meropenem trihydrate, levofloxacin, surgical operation	Improved

Splenic abscesses can be formed by hematogenous or local dissemination, which often occurs in people with immunocompromised status. The management of patients with splenic abscesses includes antibiotic therapy, percutaneous drainage, or splenectomy [21]. Percutaneous drainage is recommended when the risk of sepsis is high after splenectomy, but multilocular abscesses, intractable abscesses, and ruptured abscesses with bleeding are contraindications for percutaneous drainage [22]. In our case, the splenic abscess was a single focus, and improved after percutaneous drainage and medication without splenectomy.

In conclusion, splenic abscesses caused by *S. anginosus* bacteremia are rare, and no study has reported the urinary tract as the original focus till now. Our case expands the knowledge about the potential source of infection in such patients. Treatment of the primary disease should be instituted as early as possible. Active intervention, prevention of *S. anginosus* invasion of the blood, control, or avoidance of the occurrence or progression of the abscess can effectively improve the prognosis and increase the survival rate.
